# Impact of COVID-19 Pandemic on Respiratory Syncytial Virus (RSV) Prophylaxis Program: A Tertiary-Care Center Experience

**DOI:** 10.7759/cureus.42563

**Published:** 2023-07-27

**Authors:** Hamza M Kelabi, Adel S Alharbi, Abdullah S Alshamrani, Khaled Baqais, Ayed M Alenazi, Mansour M Alqwaiee

**Affiliations:** 1 Department of Pediatrics, Prince Sultan Military Medical City, Ministry of Defense, Riyadh, SAU

**Keywords:** pediatrics, saudi arabia, prophylaxis, rsv, covid-19

## Abstract

Objectives: The purpose of this investigation was to evaluate the effects of the COVID-19 pandemic on the respiratory syncytial virus (RSV) prevention program at our institution across three time frames: 2019-2020, 2020-2021, and 2021-2022.

Methods: We carried out a descriptive, single-site observational study spanning four years, from June 2019 to June 2022. Our study included patients in our institution's RSV program who met our enrollment criteria. We collected information about the number of children receiving immunoprophylaxis, immunoprophylaxis doses, and RSV risk factors.

Results: The number of patients receiving immunoprophylaxis dropped across the three periods, from 315 patients in the first period (2019-2020) to 176 in the second period (2020-2021), and further decreased to 128 in the third period (2021-2022). Following the COVID-19 pandemic, there was a 50% reduction in the number of patients receiving immunoprophylaxis. The proportion of RSV-infected patients remained relatively similar in the first and second periods (2.86% and 2.27%, respectively) but increased in the third period (5.47%). In the first period, most patients (60.32%) received seven doses, 11.75% got four to six doses, and 27.95% received three doses or fewer. The second period saw 59.66% of patients receiving four to six doses and 40.34% receiving three doses or fewer. In the third period, a mere 9.38% received four to five doses, while 90.63% got three doses or fewer.

Conclusions: While preventative measures associated with COVID-19 may have helped reduce the number of RSV cases, the pandemic seems to have caused a significant decrease in the number of children receiving immunoprophylaxis and the doses of immunoprophylaxis. More extensive, multicenter research is needed to understand the impact of the COVID-19 pandemic on RSV immunoprophylaxis, its activity, and seasonal patterns fully.

## Introduction

As the most common cause of bronchiolitis and viral pneumonia in children, respiratory syncytial virus (RSV) is a worldwide health problem [1]. It is a seasonal infection and the leading cause of lower respiratory tract infection (LRTI) in children less than two years old around the globe, including in Saudi Arabia [2,3]. The WHO estimates that annually 33 million individuals suffer from acute lower respiratory infection due to RSV, leading to 3 million hospitalizations and 59,600 fatalities [4]. According to a study that investigated the prevalence of RSV in Saudi Arabia between 1993 and 2018, RSV was the leading cause of acute LRTIs in children less than one-year-old. It was responsible for up to 79% of LRTIs in children under five. Although most cases occur between November and March, infections have been observed in Saudi Arabia throughout other times of the year [5].

Patients at high risk for RSV infection include those born prematurely (less than 29 weeks gestational age) and less than 12 months old [6,7]. The first year of life may be considered the most critical for preterm children with chronic lung disease caused by bronchopulmonary dysplasia [8]. Patients with congenital heart disease (CHD), neuromuscular disease, congenital anomalies, and immunocompromise status are considered high-risk [9,10]. Clinical manifestations included such as fever, respiratory symptoms (i.e., cough, shortness of breath, sore throat, and rhinorrhea), musculoskeletal (i.e., body ache and chest pain), neurological (headache and confusion), and gastroenterology (diarrhea, nausea, and vomiting) [11].

COVID-19, caused by a newly emerged novel virus, was reported in December 2019 in Wuhan as the first case. SARS-CoV-2 causes a worldwide pandemic [12-14]. Saudi Arabia began taking precautions before any confirmed cases were reported and before the WHO declared COVID-19 a worldwide pandemic [15]. The restriction and protective measures include a curfew, travel restriction, working from home, any travel history, and social distancing. A study was conducted at King Saud University Medical City to evaluate the effect of COVID-19 on the routine pediatric immunoprophylaxis program. Their findings showed a significant drop in March, April, and May 2020, 49.93%, 71.90%, and 68.48%, respectively, compared with the mean numbers of immunoprophylaxis visits during the same months from 2017 to 2019 [16].

In this study, we aimed to assess the impact of the COVID-19 outbreak on the RSV prophylaxis program at our center for three periods: 2019-2020, 2020-2021, and 2021-2022.

## Materials and methods

Study design and setting

Patient Information

This observational study was conducted over four years, from 2019 to 2022. Patients from the RSV program who fulfilled the recruitment criteria were included in this study.

The participants were recruited following strict exclusion and inclusion criteria. We included newborns having a gestational age less than 29 weeks and aged ≤12 months at the start of the RSV season, pre-term babies (29-32 week period of gestation), and infants (33-35 week period of gestation) with two or more risk factors, such as aged siblings, childcare attendance, exposure to environmental pollutants, congenital abnormality, severe neuromuscular disease, age <24 months with bronchopulmonary dysplasia, or CHD. Participants who did not meet the inclusion criteria were excluded. During the recruitment process, despite COVID-19 restrictions, we followed the Saudi Initiative of Bronchiolitis Diagnosis, Management, and Prevention guidelines [5], as shown in Table 1.

**Table 1:** The Saudi Pediatric Pulmonology Association recommendations for RSV immunoprophylaxis use across different patient categories GA: gestational age, RSV: respiratory syncytial virus, CLD: congenital lung disease, CHD: congenital heart disease

Patient Segment	Recommendations
Early preterm (<28 weeks, 6 days GA)	s12 months of age
Mid-preterm (28 weeks GA. 0 days to 32 weeks, 6 days GA)	s6 months of age
Late preterm (33 weeks, 0 days weeks GA to 35 weeks, 0 days GA)	s6 months of age at the start of the RSV season OR born during RSV season with at least one of the following risk factors: attendance at childcare, children <5 years of age who live permanently in the same household (including siblings), exposure to environmental air pollutants
Infants and children with CLD	<12 months for all; <24 months il still receiving medications for CLD within 6 months from the beginning of the epidemic season
Infants and children with hemodynamically significant CHD	<12 months for all. <24 months if still receiving medications for the cardiac condition. <6 months from the beginning of the epidemic season. Postoperative dose after cardio bypass
Children with anatomic pulmonary abnormalities or neuromuscular disorder	<24 months may be considered for infants with impaired ability to handle respiratory secretions
Immunocompromised children	<24 months may be considered for children who are profoundly immunocompromised during the RSV season
Children with Down syndrome	Recommended in children with accompanying qualifying heart disease, CLD, airway clearance issues, or prematurity (<35 weeks. 0 days GA)
Children with cystic fibrosis	<12 months with clinical evidence al CLD and for nutritional compromise <24 months with manifestations of severe lung disease OR weight for length <10^th^ percentile
Special situations: if an infant who is receiving prophylaxis experiences a breakthrough of RSV	if an infant who is receiving prophylaxis experiences a breakthrough of RSV, the monthly prophylaxis should continue as planned until a maximum of 5 doses have been administered

This study was approved by the local ethical committee of Prince Sultan Military Medical City (HP-01R079 (1503)). Signed informed consent was obtained from each participant prior to data collection. The medical records of all the subjects were collected to determine their demographic and clinical characteristics.

Data Management Plan

All experimental data, laboratory test reports, and demographic characteristics of the newborns were entered into an Excel spreadsheet (Microsoft, Washington, USA) to create a master chart. The impact of the COVID-19 outbreak on the RSV prophylaxis program at our institute was assessed over three seasons (2019-2020, 2020-2021, and 2021-2022) in terms of compliance, number of visits, doses, infections, and outcomes. Over five months, we reviewed patients' medical records under the RSV prophylaxis program for three seasons. The data were collected via an electronic health records system, which can retrieve the number of RSV prophylaxis visits to pediatric chest clinics and outside and correlate these with the consumption of RSV prophylaxis (palivizumab injection Synagis) from the pharmacy store with the RSV batch number for any child. A pediatric pulmonology physician collected the data.

Statistical Analysis

The statistical analysis was conducted using SPSS Statistics version 21.0 (IBM Corp. Released 2012. IBM SPSS Statistics for Windows, Version 21.0. Armonk, NY: IBM Corp.). Categorical data were presented as frequency and percentage. Data were visualized using the Excel Microsoft Office program.

## Results

Patients who received immunoprophylaxis

Over the three periods, the number of patients who received immunoprophylaxis was reduced from 315 patients in the first period (2019-2020) to 176 patients in the second period (2020-2021) and then declined to 128 patients in the third period (2021-2022). Post-COVID-19 pandemic, the number of patients who received immunoprophylaxis declined by 50%. Both males and females were represented in this study (Table 2).

**Table 2:** Patients who received immunoprophylaxis RSV: respiratory syncytial virus

Variables		2019-2020	2020-2021	2021-2022
Injected patients		315 (50.88%)	176 (28.43%)	128 (20.67%)
Gender	Male	142 (45.08%)	75 (42.61%)	54 (42.19%)
	Female	173 (54.92%)	101 (57.39%)	74 (57.81%)
Infected patients		9 (2.86%)	4 (2.27%)	7 (5.47%)
Adherence to RSV immunoprophylaxis	Dose 1	22 (6.98%)	17 (9.66%)	70 (54.69%)
	Dose 2	38 (12.06%)	42 (23.86%)	35 (27.34%)
	Dose 3	28 (8.89%)	12 (6.82%)	11 (8.59%)
	Dose 4	9 (2.86%)	17 (9.66%)	7 (5.47%)
	Dose 5	8 (2.54%)	39 (22.16%)	5 (3.91%)
	Dose 6	20 (6.35%)	49 (27.84%)	-
	Dose 7	190 (60.32%)	-	-

Positive RSV

The percentage of patients infected with RSV in the first and second periods was comparable (2.86% and 2.27%), respectively. However, it was slightly higher in the third period (5.47%).

Adherence to RSV doses

During the first period, most patients (60.32%) received seven doses, 11.75% received four to six doses, and 27.95% received three or fewer doses. In the second period, 59.66% received four to six doses, and 40.34% received three doses or less. In contrast, only 9.38% received four to five doses in the third period, and 90.63% received three doses or less.

Risk factors

**Figure 1:**
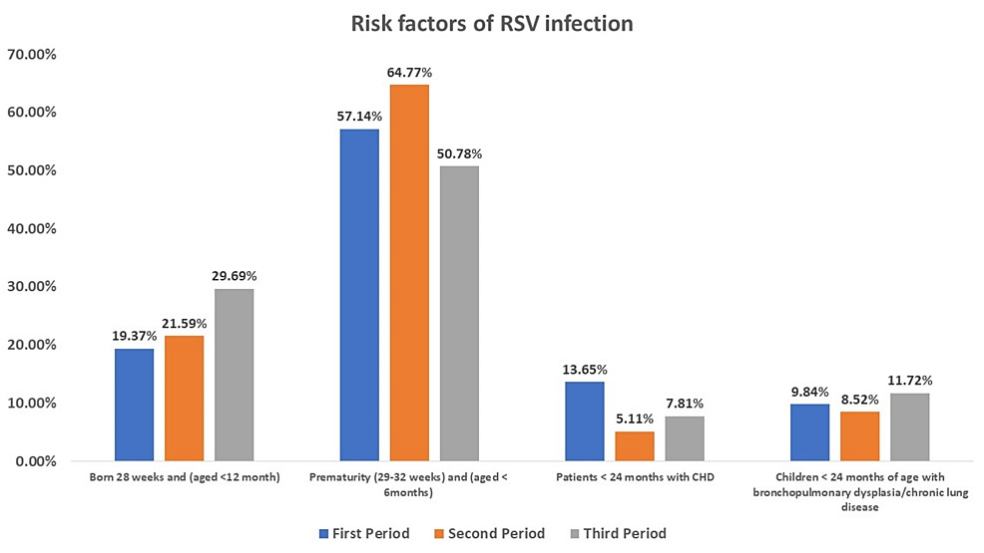
Risk factors of RSV RSV: respiratory syncytial virus, CHD: congenital heart disease

During the first period, 57.14% of the patients were premature, 19.37% were born at 28 weeks, 13.65% had CHD, and 9.84% had bronchopulmonary dysplasia or chronic lung disease. In the second period, 64.77% of the patients were premature, 21.59% were born at 28 weeks, 5.11% had CHD, and 8.52% had bronchopulmonary dysplasia or chronic lung disease. In the third period, half the patients were premature; 29.69% were born at 28 weeks; 7.81% had CHD; and 11.72% had bronchopulmonary dysplasia or chronic lung disease (Figure 1).

## Discussion

This study showed that the number of patients who received immunoprophylaxis was reduced significantly during the COVID-19 pandemic. The adherence of the patients and their families to RSV immunoprophylaxis was also negatively impacted due to the COVID-19 pandemic. Prematurity, CHD, bronchopulmonary dysplasia, and chronic lung disease were the most frequent risk factors for RSV infection.

The global spread of COVID-19 is interfering with standard child vaccination programs and putting at risk the successes in preventing vaccine-preventable diseases (VPDs) over the previous two decades [17,18]. The WHO reports that more than 80 million children, mostly in developing nations, have been affected by the widespread disruption of regular vaccination programs in at least 68 countries [19,20]. The decline in immunoprophylaxis rates for children, even for short periods during pandemics, might leave more people unprotected, increasing the likelihood of outbreaks of VPDs, including RSV, measles, polio, and pertussis [21-23]. Therefore, all nations must continue providing immunoprophylaxis services to prevent the possible spread of VPDs, some of which might have devastating consequences [24]. The pandemic hinders child vaccination programs because of several factors, including parents' and providers' fears of contracting a COVID infection, a lack of available transportation, and a focus on treating those infected with the virus [25]. The WHO stresses the need to consistently implement regular vaccination programs to guarantee optimal safety [26]. The pandemic shouldn't be taken as a reason to stop being immunized regularly; instead, it should serve as a reminder of how crucial immunoprophylaxis is as a public health policy for disease prevention [27,28].

Our findings demonstrated that the RSV infection rate slightly declined in the first year of the pandemic, then rose again. According to many studies done in the early months of the pandemic, there was a general decrease in respiratory viral infections that were not caused by COVID-19 [29,30]. Since the pandemic was announced, there has been a decrease in the number of reported flu cases worldwide [31,32]. This trend has been seen in China, Australia, and France. Parallel reductions in the occurrence of influenza and RSV were documented by Stamm et al. in Germany in 2020 and by Tempia et al. in South Africa [33,34]. The number of reported cases of influenza and RSV both decreased in the United States in 2020. It is well established that COVID-19 influences individuals' willingness to receive flu or RSV immunoprophylaxis. Additionally, simple measures like hand-washing and using masks could contribute to the reduced incidence of RSV. For the first time in 26 years of surveillance in Alaska, no children under three were hospitalized due to an acute respiratory illness for four consecutive weeks in 2020-2021 [35].

National surveys conducted in the United Kingdom and Pennsylvania, USA, found that the pandemic had encouraged previously unvaccinated individuals to get the flu vaccine [36,37]. It was proposed that the essential measures to protect against SARS-CoV-2, including frequent hand-washing, disinfection, mouth-and-nose protection, and social distancing, have contributed to the reduced rates of influenza and RSV infections during the COVID-19 pandemic [38]. On the other hand, another theory suggests that the reduced surveillance for non-SARS-CoV-2 respiratory viruses during the pandemic may also partially account for the observed decline in positive RSV cases [39]. Virus-virus interactions may also affect population-level infection dynamics through transient immune-mediated interference in individual hosts [40]. There is evidence that RSV, influenza, and human rhinovirus can inhibit one another's spread via a process known as viral interference [41,42]. The increase in RSV infections after the initial reduction could be explained by the fact that the COVID-19 control measures were eased, including the progressive re-opening of schools and shopping centers.

RSV immunoprophylaxis programs have been modified in many countries in response to the COVID-19 pandemic. In the United Kingdom, the number of doses has been increased from five to seven, and eligible children may begin the regimen as early as July rather than October [43]. The Saudi Pediatric Pulmonology Association suggests implementing home immunoprophylaxis, expanding the number of clinics and drive-through visits for the RSV immunoprophylaxis program, and promoting expedited referrals to experts [39]. Factors considered in these decisions include (1) the adverse socioeconomic sequelae of isolation/time missed from work or school, (2) more prolonged RSV hospitalizations, further straining a COVID-stricken healthcare system, and (3) increased severity of illness in vulnerable at-risk populations. Generally, in the post-pandemic period, it will be necessary to have a flexible response to RSV activity, with regular reevaluation of prophylactic recommendations by national scientific committees.

We acknowledge that our study has some limitations, including the single-center setting and lack of advanced analysis, which may hinder our findings' internal and external validity. Further, we could not perform a regression analysis to detect RSV immunoprophylaxis status change predictors due to the lack of data.

## Conclusions

In conclusion, COVID-19-associated preventive measures may have some benefits in reducing the number of RSV cases. However, a notable decline in the number of children who received immunoprophylaxis and the doses could probably be attributed to the COVID-19 pandemic. Due to the lack of supportive evidence, further multicenter studies are required to identify the impact of the COVID-19 pandemic on RSV immunoprophylaxis, activity, and seasonality.
